# Effect of Radix Stemonae concentrated decoction on the lung tissue pathology and inflammatory mediators in COPD rats

**DOI:** 10.1186/s12906-016-1444-y

**Published:** 2016-11-10

**Authors:** Zhenwei Wang, Wenlan Yang, Peilan Yang, Beilan Gao, Lei Luo

**Affiliations:** 1 Department of Respiratory, Yueyang Hospital of Integrated Traditional Chinese and Western Medicine affiliated to Shanghai University of Traditional Chinese Medicine, No. 110 Ganhe Road, Shanghai, 200437 People’s Republic of China; 2 Pulmonary Function Test Room, Shanghai Pulmonary Hospital affiliated to Tongji University, Shanghai, 200433 People’s Republic of China; 3 Department of Respiratory Medicine, Shanghai Pulmonary Hospital affiliated to Tongji University, No. 507 of Zhengmin Road, Yangpu District, Shanghai, 200433 People’s Republic of China; 4 General Outpatient Department, Community Health Service Center of Xinzhuang, Shanghai, 201199 People’s Republic of China

**Keywords:** Radix Stemonae, Chronic obstructive pulmonary disease, Pathology, Inflammatory mediators

## Abstract

**Background:**

Chronic obstructive pulmonary disease (COPD) is a common and frequently occurring respiratory disease. At present, western medicine treatment of COPD mainly focuses on symptomatic treatment. Using Chinese medicines or integrated Chinese and Western medicines to treat stable COPD has significant efficacy. In this study, we aimed to observe the effect of Radix Stemonae concentrated decoction on the lung tissue pathology and inflammatory mediators in COPD rats and explore its possible mechanism.

**Methods:**

SD rats were randomized into blank group, COPD model group and Radix Stemonae group, 10 cases in each group. Rats were fed for 112 days. Before the rats were sacrificed, lung function of the animals was tested. The right lower lung was fixed for morphologic observation. The inflammatory mediators in serum were determined using enzyme-linked immuno sorbent assay.

**Results:**

Body weight of animals in the model group was significantly decreased compared with blank group (*P* < 0.05). After gavage therapy with Radix Stemonae, body weight was significantly increased (*P* < 0.05). Compared with the blank group, pulmonary functions of rats in the model group were significantly abnormal (*P* < 0.05), while in Radix Stemonae group, these indicators turned much better than model group (*P* < 0.05). As for pathological changes in lungs, airway inflammation in the model group was aggravated. In the Radix Stemonae group, inflammation and emphysema were much milder. The concentrations of TNF-α, IL-8 and LTB4 in both model group and Radix Stemonae group were increased significantly (*P* < 0.05). But the levels in Radix Stemonae group were decreased significantly than model group (*P* < 0.05).

**Conclusion:**

Radix Stemonae concentrated decoction may mitigate and improve airway rebuilding in the lungs of COPD rats by inhibiting the release of inflammatory mediators.

## Background

Chronic obstructive pulmonary disease (COPD) is a common and frequently occurring respiratory disease, which is characterized by persistent airflow limitation [1]. It is often complicated with exacerbations and hospitalizations [2], which increased mortality and reduced life-expectancy [3]. At present, western medicine treatment of COPD, especially treatment of the stable stage, mainly focuses on symptomatic treatment [4], and drug treatment mostly focuses on improving symptoms and (or) reducing complications [5]. Using Chinese medicines or integrated Chinese and Western medicines to treat stable COPD has significant efficacy [6], improving pulmonary function and increasing diaphragm muscular tension [7]. A large amount of high-quality and high-level evidence has shown that its efficacy in improving symptoms, reducing exacerbations, and improving exercise capacity and quality of life is better than treatment with Western medicines alone [8–10].

Stemonae Radix is a traditional Chinese medicine (TCM) used as an antitussive and insecticidal remedy, which is derived from Stemona tuberosa Lour, S. japonica and and S. sessilifolia [11]. It has been widely used for the treatment of respiratory diseases in China for thousands of years. Earlier studies have shown that Stemonae Radix can release coughs [12], remove phlegm [13] and has antihelminthic, antibacterial, antituberculous and antifungal activity [14, 15]. Alkaloids are the major effective ingredients in Radix Stemonae, which includes stemoninine, stemoninoamide, bisdehydrostemoninine, neotuberostemonine, neostenine, tuberostemonine and tuberostemonine H. Liao et al. [16] found in an in vitro experiment on guinea pigs that the extract of Stemona radix had a relaxation effect on airway smooth muscle and the relaxation effect is realized by interacting with musearinic receptors and dihydropyridine binding point. Besides, the pharmacokinetics study of multiple components absorbed in rat plasma after oral administration of Stemonae radix showed that croomine and tuberostemonine would be potential bioactive components for the treatment of chronic or acute cough in rat model [17, 18].

In previous study of this study group, Radix Stemonae lung-nourishing fried paste prepared by our hospital (Hu Yao Zhi Zi Hu 05050323) was used for interference with patients with stable COPD [19]. And the results showed that Radix Stemonae lung-nourishing fried paste could mitigate the clinical symptoms of COPD patients, improve their quality of life [19]. What’s more, it is safe and reliable. However, its mechanism of action in treating COPD was unknown.

In this study, we established SD rat COPD models by exposing to cigarette smoke combined with intratracheal instillation of LPS according to the method of Yiping Song et al. [20]. Radix Stemonae concentrated decoction was used for gavage therapy. By observation of the effect of Radix Stemonae concentrated decoction on the lung tissue pathology and inflammatory mediators in COPD rats, we tried to explore its possible mechanism of action.

## Methods

### Ethics statement

All experiments in this study were approved by Ethic Committee of Shanghai University of Traditional Chinese Medicine, Shanghai, China.

### Animals

Thirty healthy male SD rats with weight of 200 ± 20 g, 10 weeks old were provided by Shanghai SLAC Laboratory Animal Co., Ltd (Shanghai, China). Rats were housed in the animal room of Shuguang Hospital 1 week before the experiment (Shanghai, China). Room temperature was maintained at 25 ± 1 °C, gas changes at 10~15 times per hour, relative humidity at (50 ± 10) %, ammonia concentration less than 14 mg/m^3^, noise ≤60 db. Sterilized diet and water were freely accessed.

### Reagents and drugs

Lipopolysaccharide (LPS) was purchased from Sigma (USA). Detection kits of TNF-α, IL-8 and LTB4 were purchased from JRDUN Biotechnology Shanghai Co., Ltd (Shanghai, China). Radix Stemonae Concentrated Decoction was obtained from Yueyang Hospital of Integrated Traditional Chinese and Western Medicine affiliated to Shanghai University of Traditional Chinese Medicine (Shanghai, China). Daqianmen cigarette (tar 13 mg/stick, nicotine 1.0 mg/stick, carbon monoxide 14 mg/stick) was purchased from Shanghai Tobacco Group Co., Ltd (Shanghai, China).

Preparation of Radix Stemonae Concentrated Decoction: It was prepared by the Department of Pharmacy of Yueyang Hospital. We boiled the raw material in water twice, concentrated the filtrate to 2 g/mL, and added 70 % ethanol for precipitation. After 24 h for standing, ethanol was removed and an appropriate amount of water was added to dilute the solution to a concentration of 0.6 g/mL. The amount needed by rats was calculated as 15 times the clinical dose for human use according to the “Dose Conversion Coefficients Table per Kilogram of Body Weight between Animals and Patients” proposed by Jihan Huang et al. in China [21] (each 250 g of Radix Stemonae Lung-nourishing Fried Paste contains Radix Stemonae crude drug 77.5 g; the clinical dose was 10 g/time, TID).

### Instruments and equipment

Microplate reader (Labsystems microplate reader, MK3), pipette (Pipetman, Gilson P), paraffin slicer (Leica, RM2235), water bath (Leica, HI1210), drying apparatus (Leica, HI1220 horizontal drying type), embedding machine (Changzhou Zhongwei Electronic Instrument Factory, BMJ-111), image analysis system (OLYMPUS, BX51), animal lung function detector (provided by the Department of Respiratory Medicine, Shuguang Hospital Attached to Shanghai University of Traditional Chinese Medicine, BioSystem XA SFT3410), electronic scales (Shanghai Precision and Scientific Instrument Co., Ltd., HANGPING FA1004N), optical microscope (OLYMPUS, BX41TF), tray electronic analytical balance (Shimadzu Corporation, AY220), electrically heated thermostatic water bath (Sumsung Laboratory Instrument Co., Ltd., DK-SD).

### Preparation of animal samples

The rats were randomized into blank group, COPD model group and Radix Stemonae group by using random number method, 10 cases in each group. Magenta (red) and picric acid (yellow) were used to mark the experimental animals. COPD model group was prepared according to the method of Song Yiping et al. [20]. LPS was injected into rat trachea at a dose of 200 ug (1 g/L) in the morning of day 1 and day 14; on days 2–13 and days 15–112, the rats were put in a self-prepared sealed organic glass poison-stained case for passive smoking, 30 min each time, twice a day at an interval of 2 h. Radix Stemonae group: the method was the same as the model group in the first 4 weeks, and from week 5 to week 16, apart from exposure to cigarette smoke, Radix Stemonae Concentrated Decoction was used for gavage every day, 3 times a day, 1 mL each time.

### Pulmonary function test

On day 112 of the experiment, we firstly performed pulmonary function tests in rats in vivo according to the method of Wang et al. [22]. Briefly, the rats were anesthetized by intraperitoneally injection of 2 % sodium pentobarbital (40 mg/kg), and fixed with a supine position on the bench. After cutting off the fur in the middle of the neck, we created an inverted “T”-shaped incision below the annular cartilage, and performed tracheal intubation and fixation. The trachea cannula was connected to a small animal spirometer to measure the ratio between forced expiratory volume at the time of 0.2 s and forced vital capacity (FEV0.2/FVC), expiratory peak flow (PEF), inspiratory resistance (Ri), dynamic lung compliance (Cldyn) for evaluation of lung function in rats.

### Morphologic observation of lung tissue

We fetched the right lower lung and put it in 4 % formaldehyde for fixation. Twenty-four hours later, the lung performed paraffin-embedding and slicing, conventional slice production, HE staining and routine pathological examination. We randomly selected 3 fields of view for each slice under the light microscope to observe the histological and morphological changes, including pathological semi-quantitative grading, judgment of the severity of lung inflammation and tracheal inflammation, and judgment of degree of alveolar fusion. Mild degree means that infiltration of lung tissue and various small bronchi by inflammatory cells contained in the lung accounts for less than 1/3 of the whole slice, and alveolar fusion accounts for less than 1/3 of the field of view of the entire slice. Moderate degree means that infiltration of lung tissue and various small bronchi by inflammatory cells contained in the lung accounts for less than 2/3 of the whole slice, and alveolar fusion accounts for less than 2/3 of the field of view of the entire slice. Severe degree means that infiltration of lung tissue and various small bronchi by inflammatory cells contained in the lung accounts for more than 2/3 of the whole slice, and alveolar fusion accounts for more than 2/3 of the field of view of the entire slice.

### Inflammatory mediators in serum and bronchoalveolar lavage fluid (BALF)

Before the animals were sacrificed, we sampled 5 mL of their abdominal aortic blood, centrifuged it at 3000 rpm for 10 min. The supernatant was sub-packed and stored at −70 °C. We cut open the chest to expose the trachea and lungs. After the right main bronchus was ligatured, the left lung was douched with 2 mL normal saline. And 1.5 mL solution (recovery rate 75 %) should be gathered each time as required, and filtered through gauze. The left lung was douched with the same method for 3 times. In total, 4.5 mL solution was recovered, of which 3.5 mL were centrifuged at 1500 rpm for 15 min, 4 °C. The supernatant were collected and stored at −70 °C. The concentrations of TNF-α, IL-8 and LTB4 in serum and BALF were determined by using enzyme-linked immuno sorbent assay according to the kit instructions.

### Statistical analysis

Statistical analyses were performed using SPSS11.5 program. Data were presented as mean ± SD. One-way ANOVA was adopted for comparison of multi-group means in line with normalized distribution and homogeneity of variance tests; Mann–Whitney was adopted for paired comparison of means not in line with normalized distribution and homogeneity of variance tests. A value with *P* < 0.05 indicated statistically significant difference.

## Results

### Comparison of general conditions

In blank group, the rats had smooth and glossy fur, bright eyes, stable breathing, normal food and water intake, good nutrition, healthy body, full muscles, flexible movements and swift response. In COPD model group, the rats became manic and upset in the early stage of exposure to cigarette smoke, and successively experienced sneezing, cough, shortness of breath and other symptoms. And in the late stage, the rats showed dry and yellow fur. Breathing was fast and shallow in some rats, slow and deep in some other rats. All rats had decreased activities, slow movements, low spirits, and reduced food and water intake. Some rats had a small amount of sticky secretions at the mouth and nose. While in Radix Stemonae group, appearance of the rats in the early stage of exposure to cigarette smoke was the same as in the COPD model group. The rats became manic and upset in the early stage of exposure to cigarette smoke, and successively experienced sneezing, cough, shortness of breath and other symptoms. After gavaged with Radix Stemonae, cough, shortness of breath and other symptoms in the rats were gradually mitigated, and mental state and behavioral activities and food and water intake were gradually normal, but their fur still remained dry and yellow.

### Body weight of rats

Body weight of the rats was measured on day 1, in week 4, 8 and 16 respectively. The results prompted that compared with the blank group, after rats were exposed to cigarette smoke for 4 weeks, body weights in the COPD model group were decreased significantly (*P* < 0.05). Compared with the model group, after gavage therapy with Radix Stemonae for 4 weeks (i.e. in week 8), body weights were increased (*P* < 0.05), as shown in Table 1.

**Table 1:** Comparison of rat weights in three groups

Group	the first day	in week 4	in week 8	in week 16
Blank	206.70 ± 4.19	267.40 ± 3.89	326.10 ± 5.30^#^	393.70 ± 3.95^#^
Model	207.50 ± 2.78	248.63 ± 3.96*	309.38 ± 5.63*	376.75 ± 5.65*
Radix Stemonae	208.20 ± 2.30	249.80 ± 4.34*	318.80 ± 5.67*^#^	387.00 ± 7.04*^#^

After One-way ANOVA, *compared with the blank group, there was significant difference, *P* < 0.05; #compared with the model group, there was significant difference, *P* < 0.05

### Pulmonary function test

The comparison of lung function in three groups was presented in Table 2. Compared with the blank group, in the COPD model group, inspiratory resistance (Ri) was increased significantly (*P* < 0.05), lung compliance (Cldyn) was decreased significantly (*P* < 0.05). The 0.2 s forced expiratory volume (FEV0.2)/forced vital capacity (FVC) and expiratory peak flow (PEF) were decreased significantly (*P* < 0.05). Compared with the model group, in the Radix Stemonae group, airway Ri was decreased significantly (*P* < 0.05). Cldyn was increased significantly (*P* < 0.05), and FEV0.2/FVC (%) and PEF were increased significantly (*P* < 0.05).

**Table 2:** Comparison of tested values of lung function in rats

Group	Number	FEV0.2/FVC (%)	PEF (mL/s)	Ri (cmH_2_O · s/L)	Cldyn (mL/cmH_2_O)
Blank	10	82.41 ± 3.68^#^	75.10 ± 2.78^#^	0.32 ± 0.02^#^	0.54 ± 0.05^#^
Model	10	64.33 ± 6.66*	55.38 ± 4.15*	0.47 ± 0.04*	0.35 ± 0.03*
Radix Stemonae	10	72.22 ± 1.16*^#^	66.87 ± 3.42*^#^	0.38 ± 0.01*^#^	0.47 ± 0.03*^#^

*Compared with the blank group, there was significant difference, *P* < 0.05. #Compared with the model group, there was significant difference, *P* < 0.05

### Pathological changes in rats

**Fig. 1:**
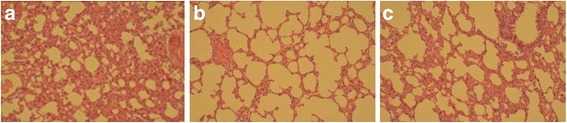
Photograph of HE-stained lung tissue under optical microscope (×100). **a** The shape and structure of rat bronchi in blank group. **b** The shape and structure of rat bronchi in COPD model group. **c** The shape and structure of rat bronchi in Radix Stemonae group

Pathological changes in lungs are shown in Fig. 1.

In blank group, the shape and structure of rat bronchi, pulmonary alveoli and alveolar septa were all normal (Fig. 1a). The structure of bronchial mucosal epithelium was intact. Ciliated columnar epithelium was regularly arranged and a few goblet cells could be seen. There were few mucosal and lamina propria cells, and no microglandular hyperplasia was observed. The size of pulmonary alveoli was normal, the structure was intact, and local invasion of a small amount of lymphocyte-dominated inflammatory cells could be seen. There was no hyperaemia and edema in alveolar septa, no significant thickening of the arterial wall, and no expansion of the alveolar cavity. In COPD model group, the structure of rat lung tissues was basically normal (Fig. 1b). Mucosal epithelium of tracheal and bronchial showed tumidness, disorder, detachment and decreased. The cilia were lodged, and increased goblet cells and gland hypertrophy were observed. Mucous plug and a large number of inflammatory cells could be seen inside the small bronchial lumen and gland catheter. Invasion of peripheral lymphocytes and plasma cells and smooth muscle hypertrophy were seen. Terminal bronchiolar mucosal epithelium was irregularly arranged. Alveolar wall turned thinner and ruptured, and alveolar turned enlargement. Besides, enlargement and mutual fusion of multiple alveolar cavities, and emphysema were observed. In Radix Stemonae group, the structure of rat lung tissues was basically normal (Fig. 1c). The symptoms of tracheal and bronchial mucosal epithelial swelling, disorder and detachment and the increasement of goblet cells were milder than those in the model group. Inflammatory cells could be seen inside the small bronchial lumen and gland catheter, and invasion of peripheral lymphocytes and plasma cells was observed, which were alleviated to different degrees compared with the model group. Hyperplasia and hypertrophy of smooth muscle was seen; alveolar wall thinning, alveolar enlargement and fusion of some alveolar cavities were all milder than those in the model group.

### Inflammatory mediators in serum and BALF

Compared with the blank control group, the concentrations of TNF-α, IL-8 and LTB4 in serum and BALF in the COPD model group and the Radix Stemonae group were increased significantly (*P* < 0.05). Compared with the model group, the levels of inflammatory mediators in serum and BALF in the Radix Stemonae group were all decreased significantly (*P* < 0.05), as shown in Tables 3 and 4.

**Table 3:** Test results of serum inflammatory mediators in rats

Group	Number	TNF-α (ng/L)	IL-8 (ng/L)	LTB4 (ng/L)
Blank	10	33.88 ± 4.90^#^	142.60 ± 19.75^#^	502.36 ± 55.62^#^
Model	10	200.43 ± 22.71*	688.78 ± 49.07*	910.94 ± 65.14*
Radix Stemonae	10	78.20 ± 22.59*^#^	378.71 ± 64.25*^#^	673.62 ± 50.82*^#^

*Compared with the blank group, there was significant difference, *P* < 0.05. #Compared with the model group, there was significant difference, *P* < 0.05

**Table 4:** Test results of BALF inflammatory mediators in rats

Group	Number	TNF-α (ng/L)	IL-8 (ng/L)	LTB4 (ng/L)
Blank	10	35.27 ± 9.16^#^	87.66 ± 23.97^#^	516.70 ± 45.34^#^
Model	10	189.90 ± 46.67*	737.25 ± 87.58*	909.27 ± 51.69*
Radix Stemonae	10	125.08 ± 29.16*^#^	254.10 ± 61.02*^#^	675.04 ± 33.82*^#^

*Compared with the blank group, there was significant difference, *P* < 0.05. #Compared with the model group, there was significant difference, *P* < 0.05

## Discussion

COPD is a common and frequently occurring respiratory disease. After an epidemiological survey among 20,245 adults in seven regions, patients suffering from COPD account for 8.2 % of the population over the age of 40 [23]. The prevalence of COPD was high, and showed an increasing trend year by year, and it was the only common disease that kept rising [24]. Risk for COPD is related to an interaction between genetic factors and many different environmental factors, which could also be influenced by comorbid disease. Environmental factors include tobacco smoking [25], infections [26], occupational dusts and chemicals [27], air pollution [28, 29] and other categories [30]. Smoking and respiratory infections are important factors in causing COPD. In this study, the method of exposure to cigarette smoke and intratracheal instillation of LPS was adopted to simulate the pathological process of COPD. In the general conditions, cough, shortness of breath and other clinical manifestations occurred, as well as weight loss. In pathological conditions, airway and lung parenchyma inflammation in the COPD model group was significantly aggravated compared with the blank group, and significant emphysema occurred. Pulmonary function test results also showed that airway resistance in the model group was increased significantly, compliance was decreased significantly, and FEV0.2/FVC and PEF were decreased significantly, suggesting that the model had been successfully established.

Abnormal expression of TNF-α, IL-8, LTB4 and other inflammatory mediators have important effects on the occurrence and acute aggravation of COPD. Inflammatory mediators can cause lung dysfunction and airway inflammation, as well as systemic inflammatory response of the body [31]. Therefore, a lot of studies [32, 33] have explored new approaches of COPD treatment by observing changes in the levels of inflammatory mediators. Among them, TNF-α showing chemotaxis effects on white blood cells, can trigger the occurrence of inflammatory response, and may participate in the formation of emphysema and damage of epithelial cells [34]. Besides, it promotes inflammatory response, tissue fibrosis, and angiogenesis in the decomposition of extracellular proteins [35]. The main function of IL-8 is activating neutrophils and release of oxygen free radicals and proteases, thereby causing injury of pulmonary alveolar epithelial and microvascular, and playing an important role in the occurrence and development of airway inflammation in COPD patients [36]. LTB4 can recruit and activate neutrophils, and further expand local inflammatory response. Meanwhile, LTB4 has an effect on increasing infiltration of inflammatory cells in bronchial mucosa [37]. After gavage therapy with Radix Stemonae, pulmonary function indicators such as expiratory peak flow (PEF), inspiratory resistance (Ri), dynamic lung compliance (Cldyn) turned much better than that in COPD model group. And the inflammation and emphysema were much milder. Inflammatory mediators (TNF-α, IL-8 and LTB4) were decreased significantly in Radix Stemonae group. Thus Radix Stemonae concentrated decoction may mitigate and improve airway rebuilding in the lungs of COPD rats by inhibiting the release of inflammatory mediators.

Radix Stemonae is the dry root of Radix Stemonae plants Stemona sessilifolia, Stemona japonica or Stemona tuberosa Lour, with a sweet-bitter state and warm nature, and having an effect on lung channel [38]. The amount of Radix Stemonae Concentrated Decoction needed by rats was calculated as 15 times the clinical dose for human use according to the “Dose Conversion Coefficients Table per Kilogram of Body Weight between Animals and Patients” proposed by Jihan Huang et al. in China [21]. But it is better to optimize the dosage for Radix Stemonae Concentrated Decoction in a pilot study.

Modern research literature reports that Radix Stemonae mainly contains alkaloids [39], including bisdehydrostemoninine, neotuberostemonine, stemoninine, stemoninoamide, neostenine, tuberostemonine and tuberostemonine H, which present therapeutic effects in lung diseases by combating bacteria, eliminating sputum, stopping cough and relaxing bronchial smooth muscle [40]. Radix Stemonae alkaloid extract has a relaxing effect on histamine-induced guinea pig bronchial smooth muscle spasm, and the effect is slow and sustained. JF Liao et al. [16] found in an in vitro experiment on guinea pigs that the water extract of Stemona sessilifolia had a spasmolytic effect on guinea pig bronchial smooth muscle and the spasmolytic effect is realized by interacting with musearinic receptors and dihydropyridine binding point.

By using ultra-performance liquid-chromatography/mass spectrometry, pharmacokinetics study of multiple components absorbed in rat plasma after oral administration of Stemonae radix showed that that croomine and tuberostemonine would be potential efficacy markers [18]. Stemoninine was identified as the major absorbed compound after oral administration of Stemonae radix detected by HPLC, which was effective for chronic or acute cough [17]. In this study, although we did not extract the active components from Radix Stemonae Concentrated Decoction, the efficacy components for COPD may be some kinds of alkaloids.

Limitations: Still, there are some limitations in the study. More groups with different dosage of Radix Stemonae should be supplemented in the study. Besides, more investigation should be provided to explore the potential molecular mechanism on treating CODP by Radix Stemonae. Thus, in the further study, we will perform more experiments to optimize the dosage for Radix Stemonae, to identify the active components and to explore its molecular mechanism.

## Conclusion

In this study, Radix Stemonae concentrated decoction was used for therapying COPD model rats. The pathological results of lung tissues showed that the manifestations of swelling, disorder and detachment in rat tracheal and bronchial mucosal epithelial and the increasing in goblet cells were improved significantly compared with COPD model group. The number of inflammatory cells in the small bronchial lumen and gland catheter was decreased than the model group. Alveolar wall thinning, alveolar enlargement and fusion were mitigated in Radix Stemonae group. The test results of inflammatory mediators in serum and BALF suggested that Radix Stemonae concentrated decoction could significantly decrease the concentrations of inflammatory mediators TNF-α, IL-8 and LTB4. Therefore, we suggested that Radix Stemonae concentrated decoction can mitigate and improve airway rebuilding in the lungs of COPD rats by inhibiting the release of inflammatory mediators, which further delay the deterioration of lung function.

## Abbreviations

Cldyn: Dynamic lung compliance
COPD: Chronic obstructive pulmonary disease
LPS: Lipopolysaccharide
PEF: Expiratory peak flow
Ri: Inspiratory resistance
